# Solvation thermodynamics from cavity shapes of amino acids

**DOI:** 10.1093/pnasnexus/pgad239

**Published:** 2023-07-26

**Authors:** Khatereh Azizi, Alessandro Laio, Ali Hassanali

**Affiliations:** The Abdus Salam International Centre for Theoretical Physics, Strada Costiera 11, 34151 Trieste, Italy; The Abdus Salam International Centre for Theoretical Physics, Strada Costiera 11, 34151 Trieste, Italy; SISSA, Via Bonomea 265, I-34136 Trieste, Italy; The Abdus Salam International Centre for Theoretical Physics, Strada Costiera 11, 34151 Trieste, Italy

**Keywords:** solvation, thermodynamics, hydrophobicity, cavity shape, amino acids

## Abstract

According to common physical chemistry wisdom, the solvent cavities hosting a solute are tightly sewn around it, practically coinciding with its van der Waals surface. Solvation entropy is primarily determined by the surface and the volume of the cavity while enthalpy is determined by the solute–solvent interaction. In this work, we challenge this picture, demonstrating by molecular dynamics simulations that the cavities surrounding the 20 amino acids deviate significantly from the molecular surface. Strikingly, the shape of the cavity *alone* can be used to predict the solvation free energy, entropy, enthalpy, and hydrophobicity. Solute–solvent interactions involving the different chemical moieties of the amino acid, determine indirectly the cavity shape, and the properties of the branches but do not have to be taken explicitly into account in the prediction model.

**Significance Statement**

Solvation is possibly the most ubiquitous and important chemical process. It drives protein–protein interactions and is at the root of most biochemical reactions. At the molecular scale, solvation requires the formation of a cavity around the host molecule. This cavity in textbooks is depicted as closely enveloping the solute. Using computer simulations, we find that the cavities surrounding the 20 amino acids in water take on branchy shapes that can extend up to 1 nm away from the solute. We show that the microscopic information of the solvation free energy, enthalpy, entropy, and hydrophobicity of all the amino acids is encoded in the shape of the cavity in which the amino acids reside.

## Introduction

Understanding the mechanism of solvation is essential in many areas of both fundamental and applied sciences (1–5), ranging from materials chemistry (6, 7) to biological processes such as protein folding and aggregation (8–12). Despite many decades of theoretical and experimental investigations, the ground rules that determine the thermodynamics and dynamical behavior of solvated systems still present unresolved and open questions (13, 14). A specific challenge in this field is bridging the gap between macroscopic and mesoscopic models, which are rather well understood, and a microscopic description, which sometimes seems to paint a more complex picture (15–18).

Molecular dynamics (MD) simulations allow for generating chemically realistic samples of different types of molecules immersed in solvent. In this regard, atomistic simulations provide a rather complex and rich understanding of the important role of solvation in both theoretical and experimental settings (19–21). For example, MD simulations have performed fairly well at reproducing properties associated with the thermodynamics of solvation such as enthalpies and entropies of solvation (22–24) and octanol–water partition coefficients (25, 26). However, the microscopic observables that enter into mesoscopic models of solvation, are typically very simple and their applicability to small and chemically complex moieties remains questionable. This is rooted in the fact that these types of models typically neglect the complexity and heterogeneity of the molecular configurations that are observed at the microscopic scale.

One such microscopic detail of solvation is that associated with the creation of a region of empty space in the liquid. Cavity formation as it is commonly referred to, is considered an essential ingredient in mesoscopic solvation models (4, 27–31). The free energy needed to form a cavity is assumed to be proportional to the volume of the cavity for small-size hydrophobic solutes and to the surface area for the large-size solutes, when surface tension starts playing a role (4). Mesoscopic models relying solely on the volume or the solvent-accessible surface area do not account for the molecular roughness and interfacial disorder which are important for small molecules (32). Indeed, we have recently found that the cavities in bulk water (33–35) as well as surrounding folded hydrophobic model polymers, for example, are highly dendritic and do not resemble quasi-spherical bubbles even beyond the nanometer length scale (18, 36). Although phenomenological corrections to the effective surface tension may account for curvature effects in these situations (17, 37), their physical applicability to chemically realistic molecules and the extent to which the cavity is fine-tuned by the molecular interactions, remain an open question.

In this work, we argue that one of the key missing ingredients of mesoscopic models in predicting solvation thermodynamics of small molecules originates from the irregular cavity shapes that host them. Using MD simulations of the 20 natural amino acids in water, we find that the cavities hosting the amino acids are highly branched. The shape of these cavities are in turn finely tuned by both the size and polarity of the chemical groups. We demonstrate that the free energy of solvation, the enthalpy, entropy, and several measures of hydrophobicity including the octanol–water partition coefficient of the amino acids in water solution, can be accurately predicted using a simple model that uses as input, *only* variables that describe the shape of the cavity. Besides including the canonical parameters such as the volume and surface area, variables that encode information about the shape of the cavity such as the lengths of the branches crucially enter into the model. The shape of the these cavities are fundamentally related to simple chemical properties of the amino acids. We believe that this observation forms a key ingredient for reconciling solvation models at the boundary between microscopic and mesoscopic scales.

## Results and discussion

**Fig. 1.**
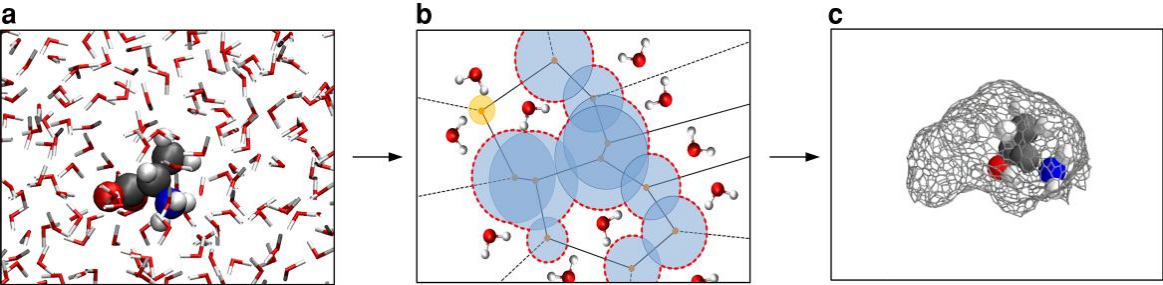
Procedure of finding the voids is illustrated. a) A snapshot of a molecular dynamics simulation showing the amino acid Alanine in a box of water. b) A simplified schematic plot of the definition of the void region in 2D, which shows the Voronoi partitioning and the empty spheres related to each Voronoi vertex. The circles with a minimum radius of 1.2 Å, and a bottle-neck of 1.1 Å are then merged to find the voids. The blue circles show the void hosting the polymer with its boundary highlighted by the red dashed line. The other Voronoi circle shown in gold is another void present in water not connected to this void. It is important to mention that for the clarity in the schematic plot, the Voronoi partitioning is illustrated considering only the oxygen atoms while the actual analysis (see Methods section) is performed including oxygen and hydrogen atoms. c) Graph representation of the surface of the void hosting Alanine from which various properties are extracted.

We performed molecular dynamics simulations of the 20 amino acids solvated in water. We then extract the cavities hosting the solute for about 1700 randomly sampled configurations for each amino acid. A schematic of the procedure used for finding the voids is illustrated in Fig. 1a-c explained in more detail in the Methods section. Starting from the molecular dynamics simulations, we remove the amino acids and subsequently conduct a Voronoi analysis that builds up the empty space using probe spheres which can then ultimately be merged to yield the void.

The shapes of the cavities are characterized using our recently developed method (36). The essential idea here is that by modeling the surface of the cavity as a graph network, we can extract various observables characterizing their shape. Besides the standard variables such as volume (*V*) and the surface area (*S*) that are used in nucleation theory (38), we can determine the longest geodesic distance of the cavity (length, *L*), the number of branches ($n_{\mathrm{br}}$), and the minimum ($d_{\min}$), maximum ($d_{\max}$), average ($d_{\mathrm{ave}}$), and total ($d_{\mathrm{total}}$) length of the branches. These noncanonical variables probe the roughness of the cavity surface as well as the extent to which the surface oscillates to and from the solute. Technical details on the simulation setup and the characterization of the voids are provided in Methods section.

**Fig. 2.**
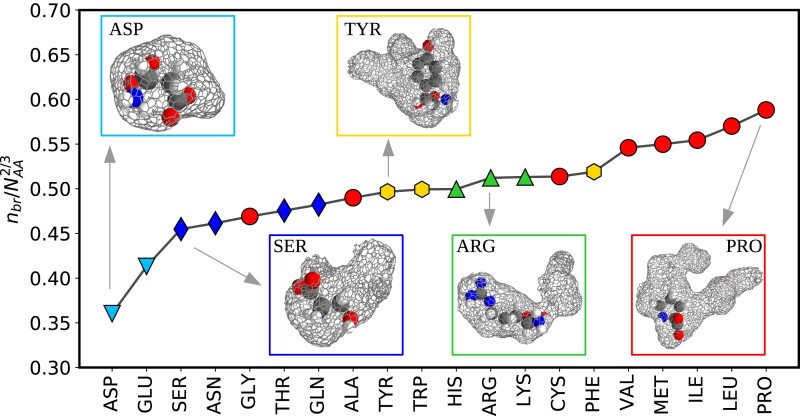
The average number of branches ($n_{\mathrm{br}}$) of the void around each amino acid solvated in water, normalized by $N^{2/3}$, that *N* is the total number of heavy atoms in the amino acid. The five groups are illustrated with different markers: nonpolar hydrophobic (red circle), polar hydrophilic (blue diamond), negatively charged (light blue down-triangle), positively charged (green up-triangle), and aromatic ones (yellow hexagon). Also one sample void for each of the groups is presented. The figure shows that the branchiness of the void differs for different amino acids, from less-branchy (for ASP) to a much more branchy voids (for PRO).

To illustrate how these quantities vary in different molecules, in Fig. 2 we show the average branch density for the 20 amino acids sorted in increasing order. The branch density is defined as the number of branches normalized by $N^{2/3}$, where *N* is the number of heavy atoms in the amino acid. For visual depiction, snapshots of the surface of the cavity surrounding different chemistries are also shown. These include, Serine (SER) which is polar, Proline (PRO) that is hydrophobic, Aspartic acid (ASP) and Arginine (ARG) that are negatively (ASP) and positively (ARG) charged respectively, and finally, Tyrosine (TYR) which is an aromatic moiety. Qualitatively, these images indicate that the topography of the cavities surrounding the amino acid is very sensitive to the underlying chemistry.

More precisely, Fig. 2 shows that the branch density varies by around 50%, from 0.35 to 0.60. The nonpolar hydrophobic amino acids tend to have more branches, while there are fewer branches around the polar and hydrophilic ones. This is consistent with the notion that hydrophobic entities tend to enhance density fluctuations by easing cavity formation (29). Curiously, positively and negatively charged groups tend to affect the properties of the branches in subtly different ways. The negatively charged amino acids, Aspartic and Glutamic acid are characterized by the lowest branch densities. This feature originates from the fact that anions tend to induce an orientational order with the protons of the water molecules closer to the charged species (39, 40). In contrast, for cations, the water dipoles arrange in a manner such that the oxygen atoms are displaced slightly further away. For the negatively charged amino acids, this feature pulls in the cavity surface closer to the solute and leads to both fewer and smaller sized branches as seen in reduced values of $d_{\mathrm{ave}}$ (see Fig. S1 in the Supplementary Material).

Fig. 2 shows that there is an apparent link between the cavity shapes and the chemistry of the amino acid. Based on these hints, we decided to attempt predicting the solvation properties of amino acids exclusively from their corresponding cavity shapes. The solvation thermodynamics of amino acids in water has previously been investigated using calorimetry experiments (41, 42) providing measurements on the free energy of solvation, as well as the contributions coming from both the enthalpy and entropy. Importantly, the solvation free energy has been shown to be accurately reproduced using molecular mechanics based models (43). In addition, there have also been numerous attempts to develop hydrophobicity scales which are inferred indirectly from thermodynamic observables such as the octanol partition coefficient using liquid chromatography (44) or the surface activity of amino acids using surface tension measurements (45). Can the shapes of the cavities shown in Fig. 2 hold *quantitative* information on these observables?

Inspired by the success of linear free energy relationships (43, 46) that are used in organic chemistry to determine correlations between reactivity and the chemical properties of molecules, we constructed a multivariate linear regression model that seeks to link the shapes of cavities to the experimental observables. Measuring solvation thermodynamics of amino acids is extremely challenging and has been the subject of several experimental and computational studies (47–50). Specifically, experimental measurements within this context are typically done on model analog compounds that most closely resemble the amino acid chemistry. Furthermore, free energies of transfer between different phases such as water and vapor, cannot be directly done for charged amino acids (51). It should also be stressed that in some experiments the thermodynamics is inferred from additive models which use a linear combination of the solvation of different molecular groups building the amino acid (41). While there appears to be more consistency in the experimental values for neutral amino acids, these problems may affect the reliability of experimental measurements of the charged ones (49). Nonadditive effects due to surface curvature have previously been noted theoretically (52) and that these effects are quite sensitive to the size of the solute (53, 54).

Given the broad spectrum of reported results, we decided to present fits including both the solvation thermodynamics for only the neutral amino acids and another, where both charged and neutral are included together. For the solvation free energy, we use the experimental data from Wolfenden (51, 55, 56). These experiments report the partition coefficients between liquid water/vapor (water/cyclohexane), as well as hydration potentials. Measurements for the amines and carboxylic acid groups need to be done in basic and acidic conditions, respectively. Knowing the respective pKas, Wolfenden reports effective partition coefficients/hydration potentials in terms of the total concentration of ionized forms of the amino acids at pH=7.

**Fig. 3.**
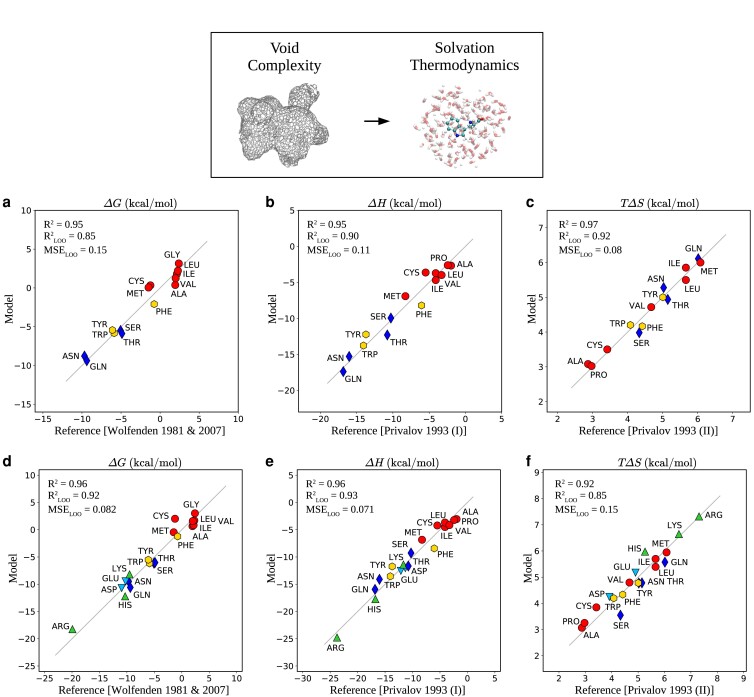
Prediction of the thermodynamic properties of solvation of amino acids in water by the shape of the cavities hosting them using linear regression analysis. On each plot, the goodness of the fits ($R^{2}$), and the LOO cross-validation results ($R_{\mathrm{LOO}}^{2}$ and ${\mathrm{MSE}}_{\mathrm{LOO}}$) are presented. a–c) The fits for the free energy, enthalpy, and entropy only for the neutral amino acids at pH=7 are illustrated whereas d–f) the fits obtained for all the amino acids are illustrated. In the case of the free energy, we use the experimental data from Wolfenden (51, 55) while the enthalpy and entropy come from Privalov et al. (41, 42).

Fig. 3a–f illustrates the quality of the prediction compared to the experimentally measured quantities. Overall, we see the quality of the fits obtained for both the neutral subset and all the amino acids are very encouraging. The variables used to train the linear models are the best combination out of the eight variables introduced earlier that quantify the shape of the void. The average values of the void variables for each amino acid are summarized in Table S1 in the Supplementary Material. Remarkably, all the thermodynamic properties we considered are predicted with high accuracy by a linear model, as seen in the Pearson correlation coefficient $R^{2}$ that are all larger than 0.9.

In order to validate the quality of our model, we also performed a leave-one-out (LOO) procedure, whereby the regression is applied to all but one amino acid. Subsequently, this new model is used to predict the properties of the amino acid that was excluded from the so-called, training set. This process is then repeated yielding an LOO prediction for all the amino acids. Although the $R_{\mathrm{LOO}}^{2}$ reduces compared to the standard $R^{2}$, the quality of the prediction is still very good.

The reported amino acid solvation energies from the Wolfenden experiments for the amino acids that would be ionized at pH=7 differ from those reported on the neutral forms of those amino acids and also experiments and theoretical predictions of model charged amino acids (41, 57). We were unsuccessful at using our cavity shapes to predict the solvation free energies for the charged groups as reported by Sitkoff et al. (57) which are a factor of up to 4 larger than the other amino acids. On the one hand, our cavity shapes come from simulations of the side chains that are charged and they appear to predict the solvation free energies derived from Wolfenden’s experiments. On the other hand, it is also clear that for charged amino acids, long-range electrostatics may not be captured by the shape of the cavity. These issues certainly require further investigation.

Looking more carefully at the components of solvation, we see that the free energy is enthalpy dominated. While there appears to be a much clearer separation between the hydrophobic and polar amino acids in the enthalpy, the same is not true for the entropy suggesting that different aspects of the void shapes control these thermodynamic quantities. The manner in which the free energy, enthalpy and entropy are manifested in the void variables is visually depicted in Fig. S2 in the Supplementary Material which represents the various coefficients of the void variables. Interestingly, our linear model recovers the competition between volume and surface area observed for the free energy and enthalpy, consistent with classical nucleation theory (38). Strikingly, we observe that the feature that the entropy is reflected most, is in the effective roughness of the void branches as seen by the dominant role of the integrated length of the branches ($d_{\mathrm{total}}$—see the unit-less coefficients in Fig. S2 in the Supplementary Material). For the exact coefficients of the linear regression, the reader is referred to Table S2 in the Supplementary Material.

The extent to which an accurate regression model can be built from the shapes of cavities has important implications on the physical chemistry of solvation. The textbook picture associated with the solvation solutes in solution is typically separated into two decoupled contributions, namely the creation of a hard-sphere cavity often interpreted as an entropic dominated term and subsequently, the reversible work that arises from turning on the interactions after inserting the solute (46). Moreover, for similar sized solutes, one might have expected similar sized cavities. Instead, the picture that emerges from our analysis is that the shapes of the cavities surrounding the amino acids are radically different. Furthermore, the specific chemical interactions between the amino acid and solvent appear leave an imprint on the properties of the cavity. Thus, the cavities surrounding the amino acids contain information on both entropic and enthalpic contributions.

The thermodynamics of cavity formation is ultimately tied to hydrophobic forces. Over the last century, there have been numerous measures developed to quantify the hydrophobicity of amino acids (58). Hydrophobicity scales are typically derived from experiments that determine the relative propensity of the amino acid to partition between different types of solvents that vary in polarity. A popular one is the hydrophobicity constant ($\pi_{Hpb}$) inferred from the octanol–water partition coefficient (44). More hydrophobic chemical moieties are likely to be more soluble in octanol rather than in water leading to a change in the partition coefficient between the two solvents. Similarly, other rankings of hydrophobicity have been derived from experiments that probe the tendency for amino acids to partition between water and cyclohexane (51, 56), as well as between the bulk and the air–water interface through surface tension measurements (45).

**Fig. 4.**
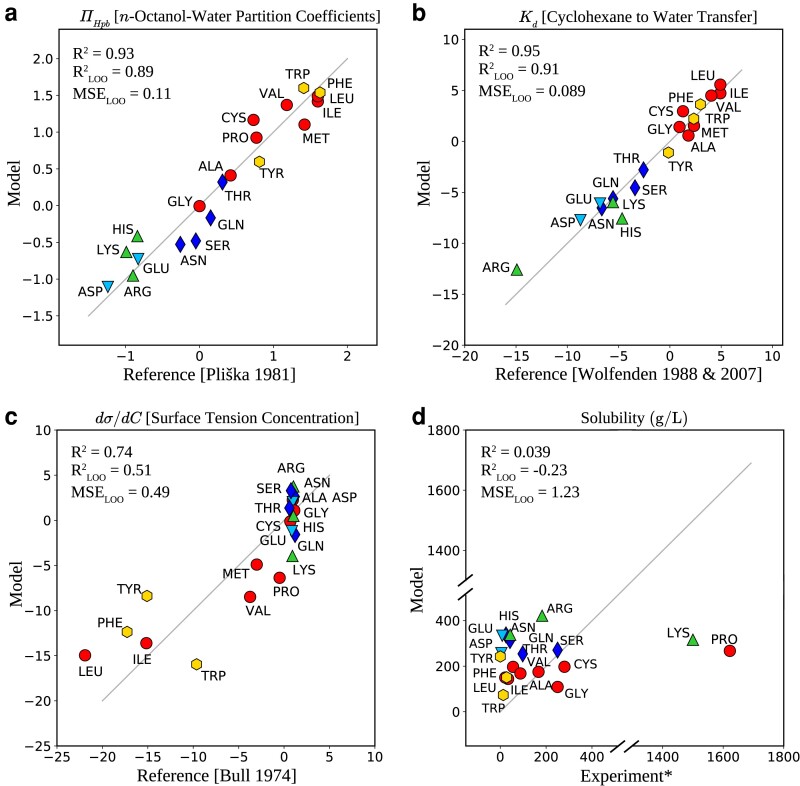
Prediction of different experimental measures (mentioned on the titles) using the variables defining the shape of the cavities around each amino acid solvated in water. a) and b) show that the cavities have all the information needed to describe the hydrophobicity constant taken from the octanol–water partition coefficient and the dissociation constant of the cyclohexane–water transfer. c) and d) On the other side, show that the prediction does not work well for the surface tension, and fails in the case of solubility. (The reference for the experimental values of a–c (44, 45, 51, 56) are given in the *x*-axis of each plot, and for the d), we have used the collected data from the CRC handbook of chemistry and physics (59).)

Given the success of our linear model at predicting the thermodynamics of solvation, we explored the possibility of identifying relationships between the shapes of the cavities and experimental classifications of hydrophobicity. The top two panels of Fig. 4 illustrates that our void variables perform extremely well ($R^{2}$ all close to 0.9) in predicting the hydrophobicity derived from the octanol partition coefficient and the $K_{d}$ between water and cyclohexane. This connection is far from trivial since the cavity shapes are derived solely from simulations of the amino acid in liquid water without any explicit knowledge of how the amino acids behave in either octanol or cyclohexane. The fact that our model works at predicting the paritioning between organic and water phases solely from cavity shapes may arise from several possibilities. On the one hand, the solutes chemistry tunes the cavity shapes in water correlate with changes in the cavity shapes in the organic phases in a similar manner. Another possibility is that the cavity shapes for the amino acids in the organic phases are not affected so much by the underlying chemistry. Examining how the cavity shapes evolve in different solvents is an interesting topic to explore in the future.

Owing to our common experience that oily objects which are hydrophobic are not soluble in water, one might intuitively expect to find a correlation between hydrophobicity and the solubility of a solute. Similarly, we would also expect that for the surface activity at the air–water interface of hydrophobic amino acids to be higher than that of polar ones. The bottom two panels of Fig. 4 show that our linear model using void variables does only moderately well ($R^{2}=0.74$) for the surface tension concentration and is completely uncorrelated with the solubility. Indeed, our results confirm that these experimental measurements probe molecular interactions, for example in the solid phase, that go beyond hydrophobic effects captured by cavity statistics of the amino acid in water. These negative results, in turn, highlight the fact that the high correlations observed for the other quantities we considered, are meaningful and nontrivial.

**Fig. 5.**
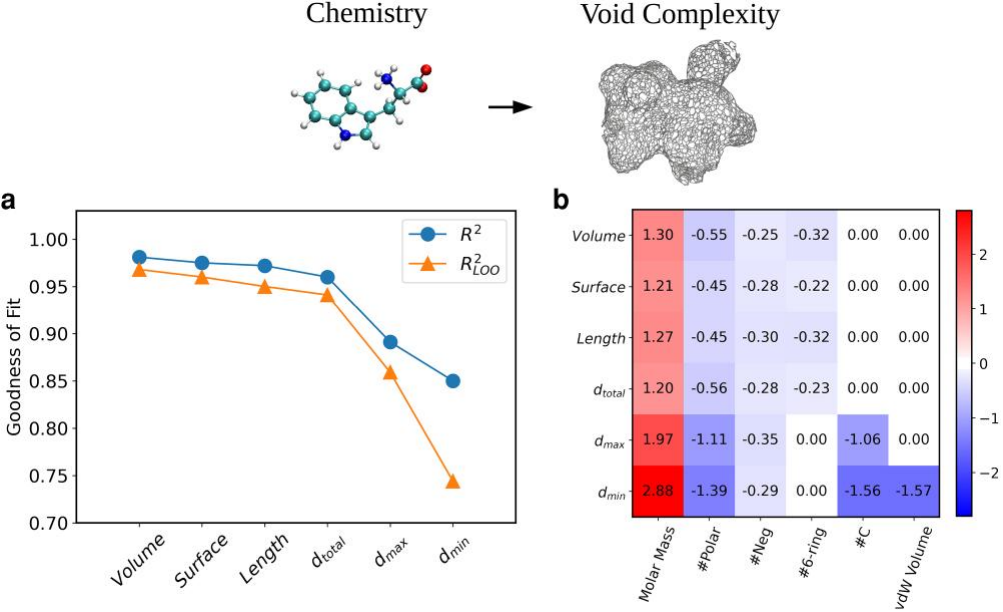
The cavities hosting the amino acids solvated in water are highly shaped and tuned by the chemical properties of the solute. a) The accuracy of the prediction of the void features ($R^{2}$) using linear regression analysis and the corresponding leave-one-out cross-validation ($R_{\mathrm{LOO}}^{2}$) results. b) The contribution of the coefficients of predicting the shape of the voids by the chemical properties of the amino acids. The plot shows the coefficients of the linear regression of predicting the standardized void variables from the standardized chemical properties. In order to have unit-less variables, and to make a meaningful comparison, we have used the standardized values, which are the values shifted to have a zero mean and then normalized by their standard deviation, as explained in the Methods section.The exact nonnormalized regression coefficients are presented in the Table S3 in the Supplementary Material.

Having shown that the shapes of cavities encode crucial information on solvation thermodynamics, we are left with one last critical step, namely to formulate how the cavities are tuned by the underlying chemistry. Fig. 2 for example, illustrates that the branch densities are likely to be modulated by the size of the amino acid as well as its polarity. Fig. 5a and b summarizes the findings that emerge from a linear model illustrating that the shape of the cavities can be accurately predicted from a set of very simple chemical variables. The corresponding correlation plots are provided in the Fig. S3 in the Supplementary Material, and the coefficients of the fit are summarized in the Table S3 in the Supplementary Material. The shape of the cavities are thus *slaved* to the underlying chemistry (20, 60). Although the simplicity of this relationship is quite remarkable, it is not unexpected. In particular, the topography of cavities are very likely tuned by the water coordination and topology of the hydrogen bond network around chemical groups of the amino acid.

An important aspect of our findings is that the shapes of the cavities are directly slaved to the underlying chemistry of the amino acids (see Fig. 5). Specifically, the zwitterionic groups do not appear to be important in predicting the properties associated with the shape of the cavity, as cavities look very similar around them. An effect of these groups may emerge due to long-range electrostatic interactions (61). To reinforce this argument, we also examined the distribution of the number of the facets (triangles making the void’s alphashape) surrounding the N and C terminal groups shown in the Fig. S4 in the Supplementary Material for a subset of amino acids (with varying polarity). Overall, we see that average number of the facets are very similar. This indicates that in our model (consistent with what is found in Fig. 5), the cavity shapes around the N and C termini of different amino acids are rather similar.

## Conclusion

In this study, we have investigated the relationship between the shape of the cavities in which solutes reside and solvation thermodynamics. The picture that emerges is poignantly simple—the complex shapes of cavities that host amino acids in solution provide a direct measure of the solvation free energy as well as its enthalpic and entropic components. The specific chemical interactions between the solute and surrounding water, ultimately affects the shape of the void that envelopes the amino acid. Recently, Havenith and co-workers have demonstrated that THz spectroscopy can be used to probe contributions coming from cavity formation and interactions between the solute and water providing insights into the competition between entropy and enthalpy (62). It would be interesting in the future to study the THz modes around amino acids with different chemistries.

Our analysis also elucidates important challenges in the interpretation of solvation thermodynamics of amino acids from experiments and at the same time, the theoretical and computational models that are used to rationalize and interpret them. We show that the cavity shapes work modestly well at predicting the solvation thermodynamics of the amino acids although there is certainly more work needed to understand for example, how pH modulates the shape of cavities and in this context, how the cavities are affected by the underlying chemistry.

The physical chemistry of cavity formation lies at the heart of fluctuations associated with hydrophobicity. Indeed, we find that cavity shapes around amino acids provide crucial microscopic information on macroscopic measures of hydrophobicity. These shapes can be inferred from simple chemistry. This contribution paves the way for investigating if this tight coupling between the shapes of cavities, the underlying chemistry, and solvation thermodynamics survives in more complex chemical systems, such as a folded protein, or a nascent crystal in water solution.

## Methods

### Molecular dynamics simulations

We performed molecular dynamics simulations for each of the 20 amino acids solvated in a cubic box of water of side length 3 nm along each direction. The amino acids are modeled using the OPLS-AA force field (63), and TIP4P-EW (64) water model. The protonation states were chosen to be those at neutral pH conditions. For the charged amino acids, one Na${}^{+}$ or Cl${}^{-}$ is added to make the system neutral. After the energy minimization, the system is equilibrated for 1 ns in the NVT ensemble and 20 ns in the NPT ensemble. The temperature and pressure is set to be 300 K and 1 atmosphere respectively, using the Nose-Hoover thermostat (65, 66) (for the NVT simulations) and Parrinello-Rahman (67) barostat (for the NPT simulations). The production simulation is then continued in the NPT ensemble for another 40 ns. All the simulations are performed using the GROMACS (68) package.

We have shown in a previous study comparing many different classical point charge water models (SPC/E, TIP4P/EW, and TIP4P/Ice) as well as the state-of-the-art many-body polarizable water model (MB-pol), that the existence of nonspherical dendritic voids is a generic property of density fluctuations in water (34). In order to assess the sensitivity of our results to the choice of the biomolecular forcefield and water model, we compared the properties of the voids obtained for 4 different amino acids with varying chemistry (1 negatively charged (Aspartic acid), 1 positively charged (Arginine), 1 hydrophobic (Leucine) and 1 aromatic (Tyrosine)) using OPLS/AA and TIP4P/EW with CHARMM (69) and TIP3P (70). The trends of the various void properties are consistently reproduced across the different forcefields as seen in the Fig. S5 in the Supplementary Material.

### Construction of voids and feature selection

We extract the voids hosting the solute for about 1700 different configurations taken from the MD simulations for each amino acid. The shape of these voids are then characterized following a procedure that was presented in a recent previous work from our group (36). Here we present a brief explanation of the procedure, and the interested reader is referred to the original reference for more details.

First, we remove the amino acid positions from the trajectory which results in the creation of a cavity. To identify this region of empty space, we consider oxygen and hydrogen of water molecules as spheres with their van der Waals radii of 1.52 Å and 1.2 Å, respectively, and mesh the space by Voronoi partitioning using the Voronota code (71). Then by setting the two parameters of the probe and bottle-neck radii to be equal to 1.2 and 1.1 Å, respectively, we extract the void. We have shown in numerous previous studies that the characterization of the voids in liquid water and hydrophobic polymers, is well captured by these parameters (18, 34). With the cavity in hand, an alphashape is constructed yielding a closed surface which can be abstracted as a connected graph.

Using the alphashape, the void is then characterized by two variables, namely, volume (*V*), and surface area (*S*). On the other hand, the graph structure allows for using graph theoretical tools to examine the topology of the void. Specifically, we find the longest geodesic distance between all two pair of nodes, and define it as the length (*L*) of the void. In addition, from the positions of both the nodes of the graph and the amino acids, we then use density-peak clustering approach (72), with a threshold cutoff being set to 5 Å, to identify branches of the void. We can then find the number of branches ($n_{\mathrm{br}}$) and the length of each branch. The minimum ($d_{\min}$), maximum ($d_{\max}$), average ($d_{\mathrm{ave}}$), and the total ($d_{\mathrm{total}}$) length of the branches, are other void variables considered as void features.

We repeated our analysis comparing how the number of branches changes for five different amino acids with different chemistries varying the threshold parameter from 3 to 7 Å. The top panel of the Fig. S6 in the Supplementary Material shows the behavior of how the average number of branches changes as a function of the threshold length. We observe that increasing the cutoff reduces the number of branches while decreasing the cutoff leads to an enhancement of the branchiness, as expected. An important outcome of the analysis is that the relative ordering of the number of branches for different chemistries is conserved as a function of different threshold parameters.

To answer the question of how the void’s shape can help in understanding the solvation thermodynamics, we built a linear model and assess the predictability of the free energy of solvation (ΔG), enthalpy (ΔH), and entropy (−TΔS) contributions, and the different hydrophobicity measures knowing the void’s variables. In this regard, we consider the average values of different void’s variables, namely: *V*, *S*, *L*, $n_{\mathrm{br}}$, $d_{\min}$, $d_{\max}$, $d_{\mathrm{ave}}$, and $d_{\mathrm{total}}$, for each of the amino acid as the possible features of the linear regression model. Looping over all the different combinations of the void’s variables, we then find the best combination that can predict each of the experimental solvation thermodynamic properties. In the Results and Discussions section, we present the important features and discuss how the solvation properties can be determined by the shape of the void around each amino acid.

Furthermore, we also performed linear regression fits for the free energy of solvation using a threshold of 4 and 6 Å which as we showed earlier, changes the absolute magnitude of the branches. The results are illustrated in the bottom left and right panels of the Fig. S6 in the Supplementary Material. The quality of the fit is almost unaffected, giving us further confidence in our results.

To emphasize the fact that the voids are not only arbitrary 3D shapes, and are indeed influenced by the chemistry of the amino acid, we do the same linear regression analysis as discussed earlier, but now to predict the void variables from the chemical properties of the amino acid. We consider 8 chemical variables, consisting: molar mass (MM), van der Waals volume (vdW), number of carbon (#C), and heavy polar atoms (nitrogen, oxygen, and sulfur) in the side chain (#Polar), the net positive (#Pos) and negative (#Neg) charge, and the number of 5-atom (#5-ring), and 6-atom (#6-ring) rings as the possible features for the linear regression model. We then loop over all the different combinations of these eight variables and predict each of the void’s variables, separately. In this regard, we are then able to answer the question of how the voids are characterized based on the chemical properties of the amino acids and which of those properties are important to determine the shape of the void. The outcome is discussed in the Results and Discussion section.

### Linear model and the cross-validation approach

We used linear regression analysis with multiple variables for both predicting the solvation properties by the void variables, and predicting the void variables by the chemical properties. To have a simplified notation, we use matrix X, and vector y to denote to the feature space, and the targets, separately. All the features in X, as well as y, are standardized before doing the regression analysis, meaning that they are shifted to zero mean, then normalized by their standard deviation. This makes all the variables being unit less, and will also result in a more reliable comparison between the coefficients. The linear regression predicting y using X is defined as: $y=\sum_{j}w_{j}X_{j}$, in which $w_{j}$’s are the weights, and *j* is the index of feature *j*.

To validate the model, one has to check its accuracy for predicting the training data set, its ability to also predict the test data, and in addition checking for over-fitting issue. To find the accuracy of the model, we first do the linear regression analysis using all the 20 data points of amino acids and find the coefficient of determination, also called as *R*-squared ($R^{2}$). The $R^{2}$ is defined as below:


(1) $$R^{2}=1-\frac{\sum_{i}(y_{i}-{\hat{y}}_{i}{)}^{2}}{\sum_{i}(y_{i}-\bar{y}{)}^{2}},$$

in which $y_{i}$, and $\hat{y_{i}}$ are the actual and the predicted value of the data point *i*, and $\bar{y}$ is the average of the actual values. The more $R^{2}$ is closer to 1, the better the linear model has been able to predict the training data. To test the model’s ability to predict unknown data points, as well as to check of the over-fitting issue, we then do the LOO cross-validation as explained in the following.

We do LOO cross-validation to assess the model’s ability to predict the test data points. In this regard, one at a time, we remove one of the data points (one amino acid) from the training data set, and build the model with the remaining 19 points, then predict the test point from the model. This process is repeated 20 times, until all the amino acids are considered once as a test data point. At each iteration, we find the mean squared error (MSE) of predicting the test data point, defined as ${\mathrm{MSE}}_{k}=(y_{k}-{\hat{y}}_{k}{)}^{2}$, with *k* to be the index of the iteration. The average of all the 20 ${\mathrm{MSE}}_{k}$ is called as ${\mathrm{MSE}}_{\mathrm{LOO}}$. Closer the average error is to zero, the more the model is able to predict the test data points. Also we define $R_{\mathrm{LOO}}^{2}$, that is calculated by Equation 1, except that now the $\hat{y_{i}}$ is the predicted values of the test data points in the LOO cross-validation procedure.

Since the error of the fit essentially grows as $\frac{1}{\sqrt{N}}$, with *N* being the number of data points, we also examined how the error in our fitting changes when we perform in addition to LOO, a leave-two-out and leave-three-out. Fig. S7 in the Supplementary Material shows on the *y*-axis, the ratio of Error${}_{i}\times \sqrt{N-i}$ to Error${}_{1}\times \sqrt{N-1}$, with *i* being the number of data points excluded from the fit. This ratio essentially quantifies how much the numerical error we obtain from the leave-two-out and leave-three-out deviates from the expected behavior. We see clearly that this ratio is close to 1 which in turn gives us confidence in our error estimate.

## Supplementary Material

pgad239_Supplementary_Data

## Data Availability

All data are included in the manuscript and/or Supplementary Material.
