# Supplementary material for: Solvation thermodynamics from cavity shapes of amino acids
Source: PNAS Nexus. 2023 Jul 26;2(8):pgad239. doi: 10.1093/pnasnexus/pgad239 (PMC10400782; doi:10.1093/pnasnexus/pgad239)
Supplement: pgad239_Supplementary_Data [file pgad239_supplementary_data.pdf]

Supplementary Information
Solvation Thermodynamics From Cavity Shapes of Amino Acids

Khatereh Azizi,[†] Alessandro Laio,[‡,†] and Ali Hassanali[*,†]

[†] *The Abdus Salam International Centre for Theoretical Physics, Strada Costiera 11, 34151 Trieste, Italy*

[‡] *SISSA, Via Bonomea 265, I-34136 Trieste, Italy*

E-mail: ahassana@ictp.it

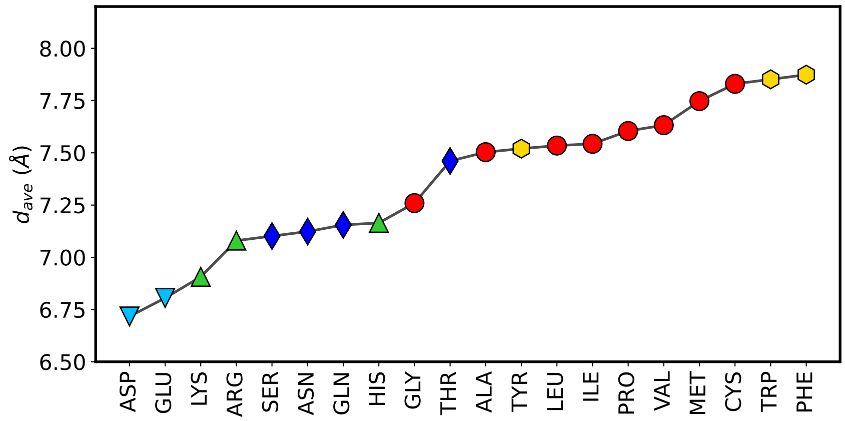

Figure S1: The average length of the branches for the 20 amino acids solvated in water, sorted in increasing order. The two negatively charged amino acids (ASP and GLU) have the lowest average branch length.

Table S1: The ensemble average of the void properties for the 20 amino acids solvated in water.

	$< V >$ (Å^3)	$< S >$ (Å^2)	$< L >$ (Å)	$< n_{br} >$	$< d_{total} >$ (Å)	$< d_{max} >$ (Å)	$< d_{min} >$ (Å)	$< d_{ave} >$ (Å)
ALA	119.002	202.878	15.373	1.618	12.119	8.111	6.911	7.503
ARG	249.629	342.269	20.463	2.685	18.877	8.462	5.872	7.080
ASN	156.119	245.164	16.938	1.997	14.129	8.011	6.270	7.123
ASP	109.896	190.666	14.817	1.559	10.371	7.233	6.214	6.717
CYS	152.693	239.860	16.782	1.880	14.632	8.701	6.979	7.830
GLN	190.981	278.890	18.036	2.238	15.922	8.244	6.159	7.155
GLU	143.758	225.528	16.238	1.918	12.971	7.644	5.998	6.805
GLY	83.080	164.465	13.745	1.372	9.978	7.601	6.914	7.259
HIS	203.264	292.337	18.584	2.319	16.501	8.290	6.132	7.165
ILE	227.382	303.005	18.845	2.399	17.930	8.791	6.392	7.543
LEU	230.806	307.185	18.956	2.467	18.396	8.835	6.340	7.535
LYS	213.449	288.992	18.645	2.382	16.361	8.040	5.863	6.906
MET	231.666	312.477	19.421	2.379	18.277	8.986	6.615	7.747
PHE	269.671	359.833	20.571	2.721	21.308	9.334	6.537	7.874
PRO	194.220	283.412	18.209	2.353	17.809	8.813	6.463	7.604
SER	116.044	202.386	15.328	1.664	11.766	7.730	6.485	7.102
THR	154.656	242.653	16.820	1.902	14.048	8.314	6.637	7.461
TRP	318.277	412.452	21.968	3.037	23.643	9.495	6.450	7.852
TYR	260.775	350.904	20.384	2.746	20.541	8.955	6.226	7.520
VAL	194.640	275.321	17.907	2.184	16.585	8.750	6.606	7.632

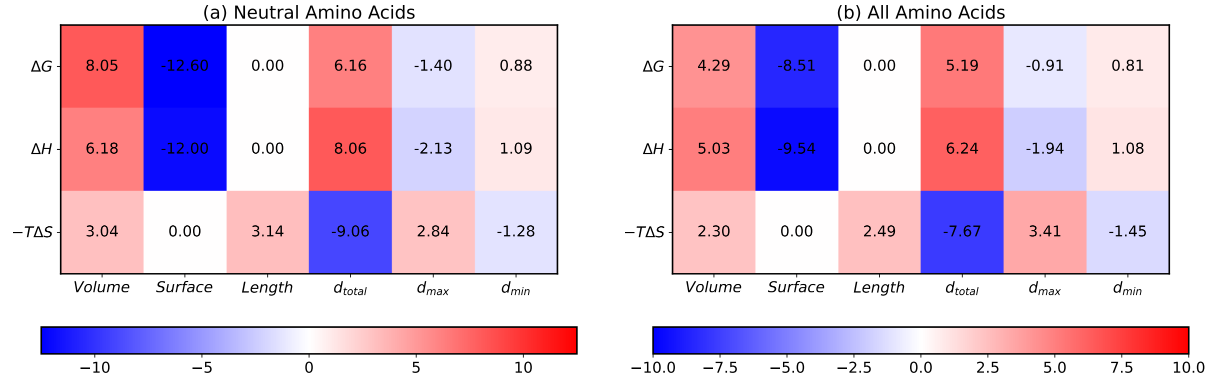

Figure S2: The contribution of each void variable in the solvation energies. The plot shows the coefficients of the linear regression of predicting the standardized solvation energies from the standardized void variables. In order to have unit-less variables, and to make a meaningful comparison, we have used the standardized values, which are the values shifted to have a zero mean and then normalized by their standard deviation, as explained in the Methods section.The exact non-normalized regression coefficients are presented in Table S2.

Table S2: Coefficients of the linear regression of predicting the solvation energies from the void properties. Please note that the coefficient related to each variable is based on the unit presented in parenthesis for that property.

Solvation Energy (kcal/mol)	Constant (kcal/mol)	$< V >$ (Å^3)	$< S >$ (Å^2)	$< L >$ (Å)	$< d_{total} >$ (Å)	$< d_{max} >$ (Å)	$< d_{min} >$ (Å)
ΔG (neutrals)	1.694	0.527	-0.807	0.000	6.903	-10.700	14.872
ΔH (neutrals)	8.563	0.546	-1.041	0.000	12.142	-21.977	24.046
$-T\Delta S$ (neutrals)	-5.085	0.0543	0.000	1.709	-2.763	5.950	-5.692
ΔG (all)	-18.930	0.418	-0.806	0.000	8.532	-9.107	15.023
ΔH (all)	15.401	0.520	-0.959	0.000	10.721	-19.618	20.746
$-T\Delta S$ (all)	-10.887	0.0473	0.000	1.490	-2.616	6.833	-5.541

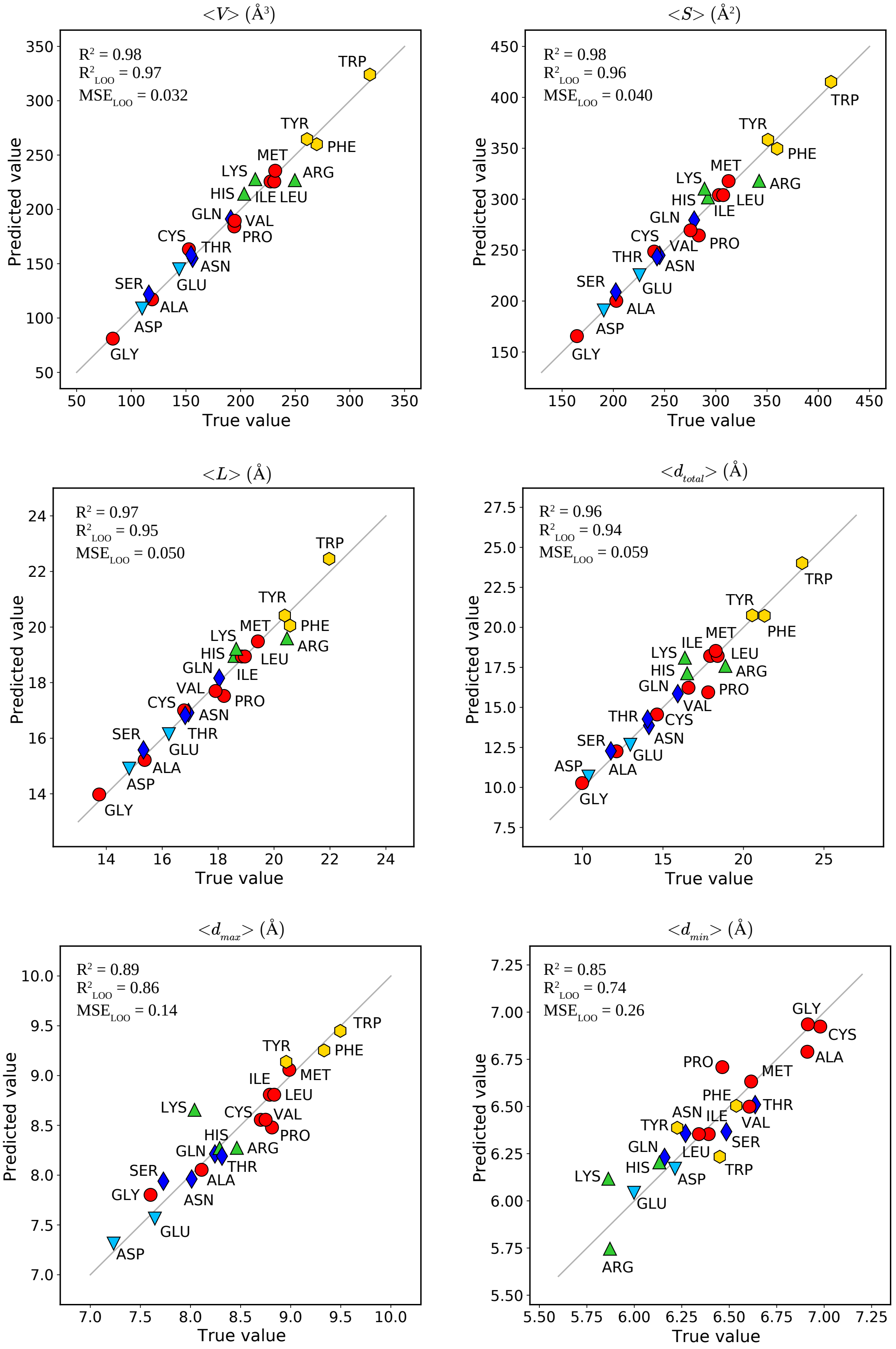

Figure S3: The correlation plots of the prediction of void variables from the chemical properties of the solvated amino acid. The figure shows that the void features are highly tuned by the chemical properties of the solute as seen from the high values of R^2 and R^2_{LOO} from the linear regression analysis.

Table S3: Coefficients of the linear regression of predicting the void properties from the chemical natures of the amino acids. Please note that the coefficient related to each variable is based on the unit presented in parenthesis for that property.

	Constant	Molar Mass (gr/mol)	#Polar	#Neg	#6-ring	#C	vdW Volume (Å^3)
$< V >$ (Å^3)	-112.433	2.578	-36.559	-48.790	-53.364	0.000	0.000
$< S >$ (Å^2)	-19.639	2.468	-30.729	-56.555	-38.359	0.000	0.000
$< L >$ (Å)	7.336	0.0885	-1.0514	-2.102	-1.898	0.000	0.000
$< d_{total} >$ (Å)	-0.357	0.142	-2.239	-3.333	-2.306	0.000	0.000
$< d_{max} >$ (Å)	4.923	0.0384	-0.728	-0.686	0.000	-0.287	0.000
$< d_{min} >$ (Å)	5.538	0.0302	-0.491	-0.307	0.000	-0.226	-0.0180

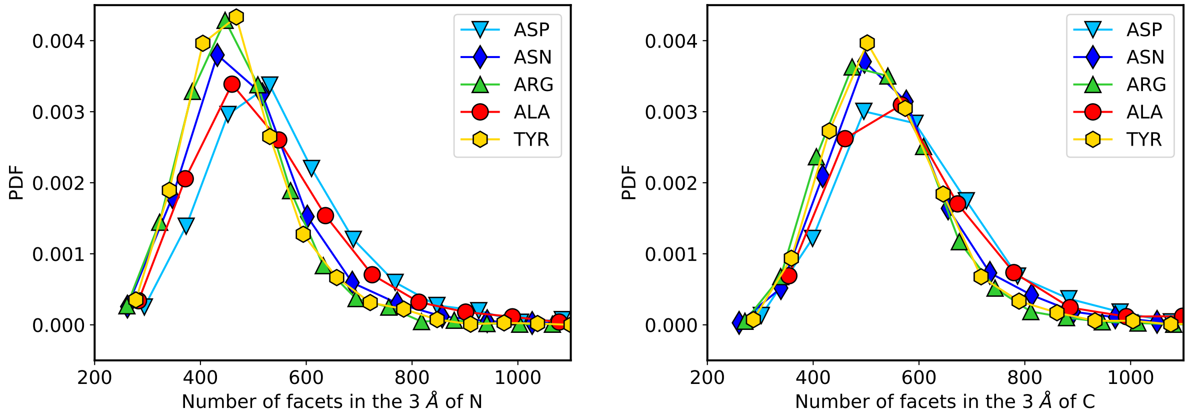

Figure S4: The similarity of the void properties around the backbones of the amino acids. The number of triangles (facets) of the alphashape creating the surface of the void in the 3Å distance from N and C termini. The plot is shown for a sample of five amino acids with different chemistry.

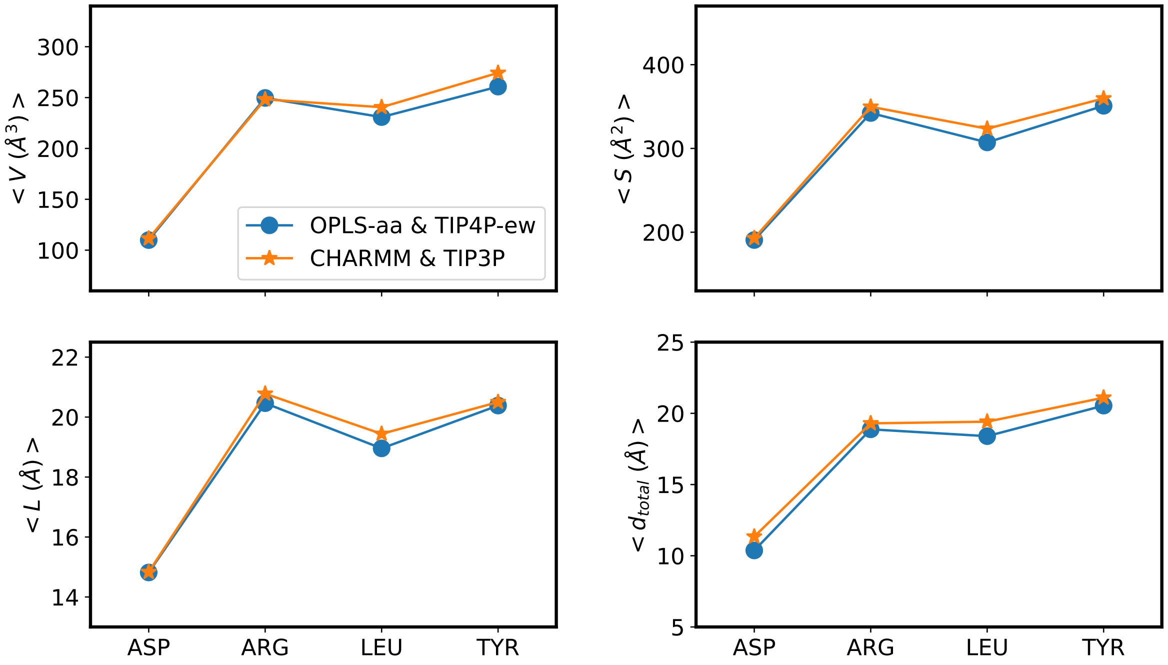

Figure S5: The effect of choosing force field on the void properties. The plot shows that void variables are very similar when using the OPLS-aa and TIP4P-ew (the manuscript), with CHARMM and TIP3P, which shows the robustness of the results when choosing an appropriate force filed.

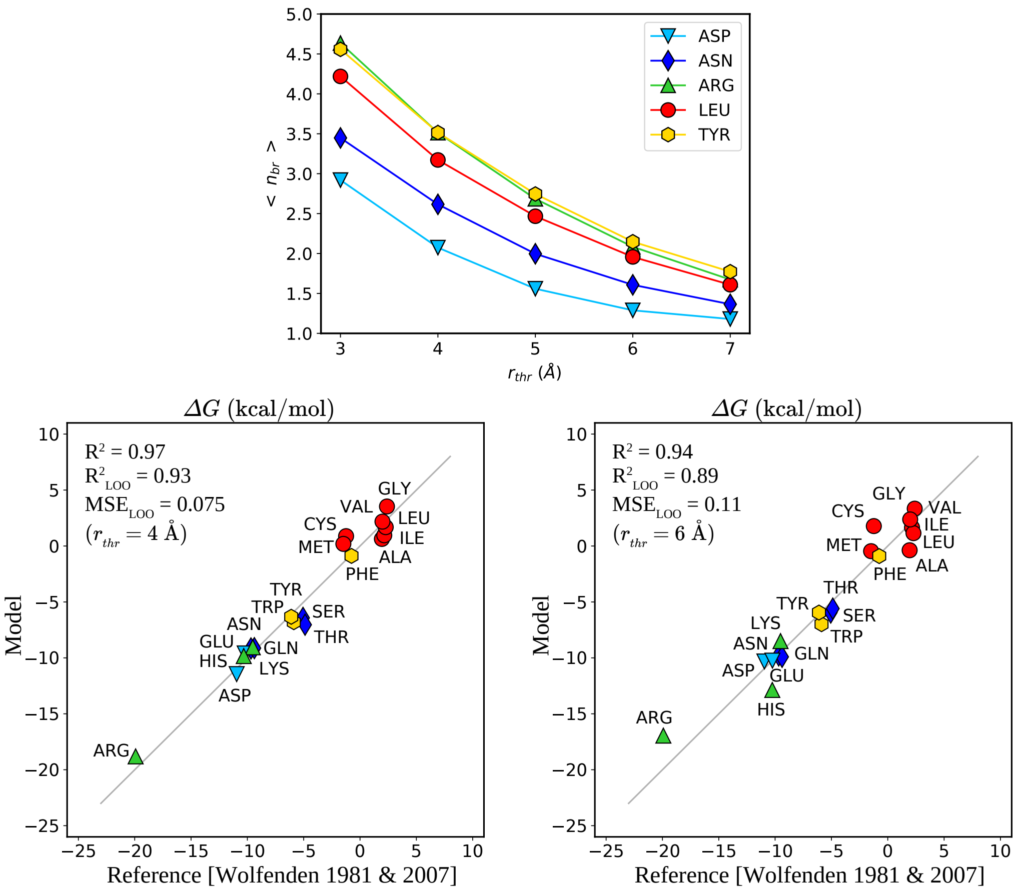

Figure S6: The effect of the chosen threshold cutoff in the clustering approach for defining the branches. The top panel shows that by increasing the threshold, the number of branches decrease, as expected. The two bottom panels shows that the linear regression of predicting the free energy of solvation using two threshold cutoffs (4 and 6 Å) is still valid and does work reasonably well.

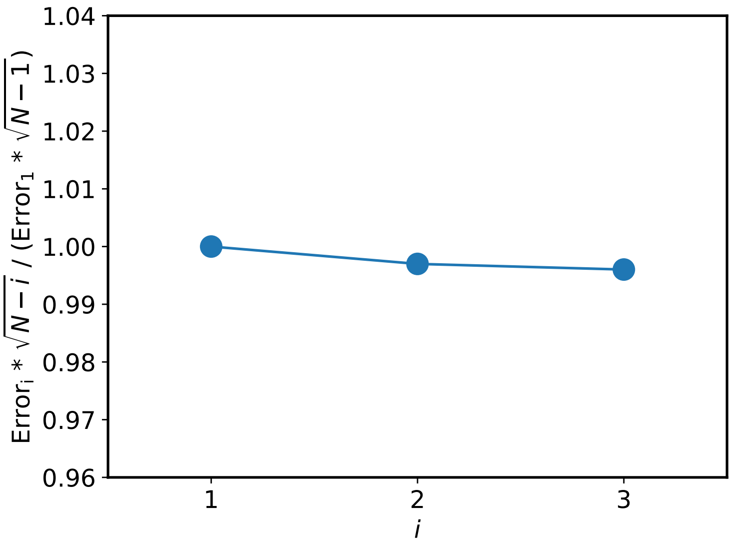

Figure S7: The scaling of the error in the linear regression in leave- one, two, and three out cross validation. The plot shows that the error scales, as expected, by $1/\sqrt{N-i}$, with N and i being the total number of data points, and the data points left out, respectively. The value of around 1 for the y-axis confirms the validity of the method.
